# Mitochondria-targeted strategies in cancer radiotherapy: from ROS regulation to immunogenic cell death

**DOI:** 10.3389/fcell.2026.1861854

**Published:** 2026-06-15

**Authors:** Jiamin Guo, Qing Xin, Zheran Liu, Shuang Dai, Jiacheng Li, Yu Min, Zhigong Wei, Yuzhu Hu

**Affiliations:** 1 Department of Biotherapy, Cancer Center, West China Hospital, Sichuan University, Chengdu, Sichuan, China; 2 Nuclear Physics and Medical Research Key Laboratory of Sichuan Province, Sichuan University, Chengdu, Sichuan, China; 3 Health and Management Center, General Practice Medical Center, West China Hospital, Sichuan University, Chengdu, China; 4 College of Medical and Life Sciences, Chengdu University of Traditional Chinese Medicine, Chengdu, Sichuan, China; 5 Department of Radiation Oncology, State Key Laboratory of Biotherapy and Cancer Center, West China Hospital, West China Medical School, Sichuan University, Chengdu, China

**Keywords:** immunogenic cell death, mitochondria, radiotherapy, Radiotherapy–immunotherapy synergy, reactive oxygen species

## Abstract

Radiotherapy (RT) is a crucial treatment modality for various solid tumors. Its antitumor effects not only depend on direct DNA damage but also involve the disruption of redox homeostasis centered around mitochondria and the activation of immune responses. Mitochondria, as a central hub for cellular energy metabolism and apoptosis signaling, serve both as the primary source of reactive oxygen species (ROS) and as key targets for ROS attack during RT. RT promotes the sustained accumulation of mitochondrial ROS (mROS) through mechanisms such as damage to the mitochondrial respiratory chain complexes, while also triggering compensatory clearance responses from antioxidant systems. The dynamic balance between these processes determines the cell’s fate. Moreover, mitochondria play a crucial regulatory role in immunogenic cell death (ICD). Damage-associated molecular patterns (DAMPs) such as mitochondrial DNA (mtDNA), ATP, and cytochrome c, along with ROS-mediated endoplasmic reticulum stress and calcium signaling, promote dendritic cell (DC) maturation and induce antitumor immunity. In recent years, mitochondria-based RT sensitization strategies have emerged, including small molecules that modulate mROS production or antioxidant defenses, mitochondria-targeted nanocarriers, gene-editing approaches, and bioregulatory or metabolic interventions. Most nanoplatforms, gene-editing strategies, and ICD-amplifying approaches are currently supported mainly by preclinical evidence, where they have been shown to enhance RT-induced tumor cell death, promote DAMP release, and improve antitumor immune activation. In contrast, selected metabolic or bioregulatory interventions have begun to enter early clinical evaluation, including ketogenic dietary interventions combined with immune checkpoint blockade in metastatic melanoma and renal cell carcinoma (e.g., NCT06391099 and NCT06896552), although definitive evidence of clinical benefit in combination with RT remains limited. This review systematically discusses mitochondrial mechanisms of ROS generation and clearance during RT, mitochondrial regulation of ICD pathways, and emerging mitochondria-targeted radiosensitization strategies, while emphasizing the current distinction between preclinical promise and clinical translational maturity.

## 1 Introduction

Radiotherapy (RT) is one of the main treatment modalities for nearly all types and stages of malignant tumors (Forenzo and Larsen, 2024). The effects of ionizing radiation (IR) on cellular components, particularly DNA damage formation, have been extensively studied. After irradiation, DNA damage can occur through two distinct mechanisms: (i) direct effects, which involve the direct ionization of DNA molecules; and (ii) the excitation and ionization of water, the most abundant component within cells, resulting in the generation of various reactive oxygen species (ROS). These ROS then attack other critical molecules, including nucleic acids, proteins, and lipids, causing oxidative damage (Pouget et al., 2018; Sonveaux, 2017). This represents one of the primary mechanisms by which RT kills tumor cells. However, ROS-mediated oxidative stress lacks specificity, and when accumulated at high doses, it can also damage surrounding normal tissue cells, leading to both acute and chronic radiation-induced injuries (Smirnova et al., 2018; Tamarat et al., 2024). Therefore, accurately regulating the spatiotemporal distribution and levels of ROS is a key issue for enhancing the efficacy and safety of RT.

Mitochondria are the primary sites of cellular aerobic respiration, adapting to the demands of rapid tumor growth by regulating various energy-producing processes (Missiroli et al., 2020). Beyond functioning as cellular powerhouses, mitochondria are also the main organelles responsible for intracellular ROS production and for maintaining the balance between oxidative stress and inflammation (Kuznetsov et al., 2022). Mitochondria represent one of the most critical targets of RT-induced damage. Alterations in mitochondrial size and shape, or mutations in mitochondrial DNA, disrupt normal physiological functions, thereby enhancing radiation adaptation (Bian et al., 2022). Under radiation exposure, mitochondria engage in multiple signaling pathways, including DNA damage response, cell cycle regulation, cell death (e.g., apoptosis, ferroptosis), and immune activation (Averbeck and Rodriguez-Lafrasse, 2021). Mitochondrial damage can lead to further ROS accumulation, forming a positive feedback loop that amplifies oxidative stress and ultimately triggers the mitochondrial apoptotic pathway (Burke, 2017). In addition, mitochondria can induce intrinsic apoptosis through the release of cytochrome c and other pro-apoptotic factors. These processes are essential for effective tumor cell eradication (Burke, 2017).

However, mitochondria are not only key metabolic sites that regulate tumor initiation and progression, but also influence the efficacy of RT through multiple mechanisms. Existing studies have shown that mitochondrial functional alterations contribute to resistance in head and neck squamous cell carcinoma and glioma (Grasso et al., 2020; Gao et al., 2020). The radiosensitivity of other common solid tumors, such as non-small cell lung cancer, breast cancer, and pancreatic cancer, is also closely linked to various mitochondrial metabolic pathways, including the regulation of oxidative stress and participation in oxidative phosphorylation (Schoenfeld et al., 2017; Benej et al., 2018; Fisher and Goswami, 2008). Moreover, tumor cells exhibit features such as mitochondrial metabolic reprogramming and the active ROS scavenging system, which allow them to evade immune recognition and escape cell death signals (Benej et al., 2018; Liu et al., 2025). Therefore, targeting mitochondrial function by modulating ROS metabolism to restore sensitivity to radiation has emerged as a promising strategy to improve radiotherapy outcomes. Interventions in mitochondrial function, such as inhibiting the respiratory chain, blocking oxidative phosphorylation, disrupting ROS homeostasis, or promoting mitochondria-mediated cell death pathways, can not only enhance RT-induced cytotoxicity but also induce immunogenic cell death (ICD), leading to the release of tumor-associated antigens and the activation of both innate and adaptive immune responses. This ultimately enables synergistic effects between radiotherapy and immunotherapy (Xu et al., 2025). Such strategies provide new models for basic researchers to explore mechanisms of radioresistance and offer novel therapeutic targets for clinicians, with the potential to improve the overall efficacy of RT and long-term survival rates in patients with solid tumors.

**FIGURE 1:**
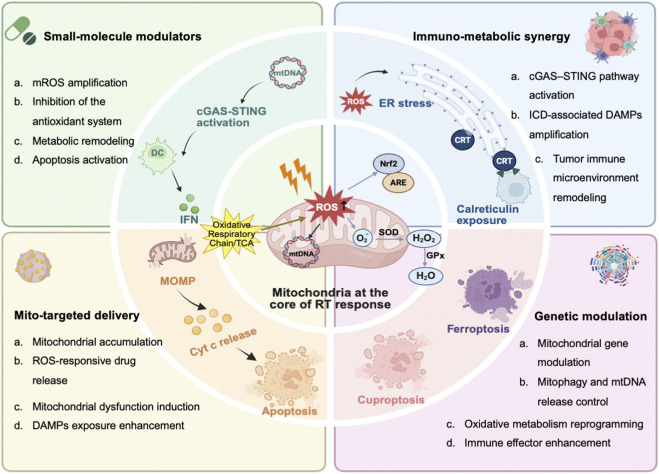
The structure diagram of this study.

This review will systematically summarize the mechanisms of mitochondrial-mediated ROS generation and scavenging during tumor RT, as well as the mitochondrial-regulated pathways of ICD. It further discusses cutting-edge strategies targeting mitochondria through small molecules, nanomaterials, gene editing, and biological approaches to enhance radiotherapy efficacy and antitumor immunity (Figure 1).

## 2 Mitochondria-mediated mechanisms of ROS generation and scavenging during RT

### 2.1 Mechanisms of ROS generation

ROS are highly reactive oxygen-derived molecules naturally produced during cellular aerobic metabolism (Cheung and Vousden, 2022), They are considered byproducts of aerobic respiration and are primarily generated within mitochondria. Among them, the superoxide anion (O_2_^−^), hydrogen peroxide (H_2_O_2_), and hydroxyl radical (·OH) are the most common in biological systems (Murphy et al., 2022) These molecules readily engage in redox reactions, inducing oxidative modifications of biomacromolecules and thereby participating in cellular redox signaling and biological functions (Sies et al., 2022). In addition to mitochondria, the endoplasmic reticulum (ER) represents another important source of ROS. Within the ER, ROS are mainly produced in the form of H_2_O_2_, accounting for approximately 25% of total cellular ROS production (Hirano et al., 2021).

During normal oxidative phosphorylation, approximately 1%–5% of electrons “leak” from the mitochondrial respiratory chain to oxygen molecules, generating ROS (Azzam et al., 2012). Under basal conditions, this process is maintained at a relatively low level as part of cellular regulation. However, following IR, this “electron leakage” effect is markedly amplified, leading to elevated intracellular ROS levels. As IR penetrates cellular tissues, it can directly ionize biomacromolecules such as DNA, proteins, or lipids, but more commonly it ionizes intracellular water molecules, thereby producing a large number of primary free radicals. These radicals possess extremely high chemical reactivity and rapidly attack critical intracellular targets, with mitochondria being among the most vulnerable organelles (Saini and Gurung, 2025). Mitochondrial DNA (mtDNA), lacking protective histones and possessing limited repair capabilities, is highly susceptible to ROS attacks, leading to mutations, breaks, or loss of function (Caito et al., 2015). At the same time, the polyunsaturated fatty acids present in the mitochondrial membrane are prone to lipid peroxidation, which disrupts membrane integrity and mitochondrial membrane potential, thereby further aggravating mitochondrial dysfunction (Caito et al., 2015). Primary ROS generated by IR can also impair the structure and function of mitochondria and respiratory chain complexes, which in turn further increases ROS production, creating a vicious cycle (Saini and Gurung, 2025).

### 2.2 Amplification of mitochondrial ROS after irradiation

In normal cells, mitochondrial ROS (mROS) levels are tightly regulated (Averbeck and Rodriguez-Lafrasse, 2021; Burke, 2017). However, due to metabolic reprogramming and enhanced oxidative phosphorylation activity, tumor cells generally harbor a higher mitochondrial content and elevated basal ROS levels, rendering them more sensitive to additional oxidative stress (Glorieux et al., 2024). As noted earlier, radiotherapy can directly damage mitochondria and respiratory chain complexes, particularly complex I, resulting in impaired electron transfer and excessive electron leakage. Under combined stress signals, including mitochondrial membrane potential disruption, calcium homeostasis imbalance, and altered membrane permeability, mitochondria can generate a “second wave” of ROS within hours after irradiation. Additionally, compared to conventional X-rays, high-energy particles such as carbon ions exhibit a higher linear energy transfer (LET), enabling them to generate denser ionization tracks along their path within cells. This facilitates more direct disruption of mitochondrial structures and induces more intense mROS production (Jin et al., 2018a; Jin et al., 2018b). This enhanced oxidative stress can cause a rapid collapse of mitochondrial membrane potential, release of cytochrome c, and activation of downstream caspase pathways, ultimately driving apoptosis. Therefore, the amplification of mROS is considered one of the key mechanisms by which RT induces apoptosis, necrosis, or programmed cell death (such as ferroptosis) (Chipuk et al., 2021; O'Malley et al., 2020).

### 2.3 ROS clearance and antioxidant defense

**FIGURE 2:**
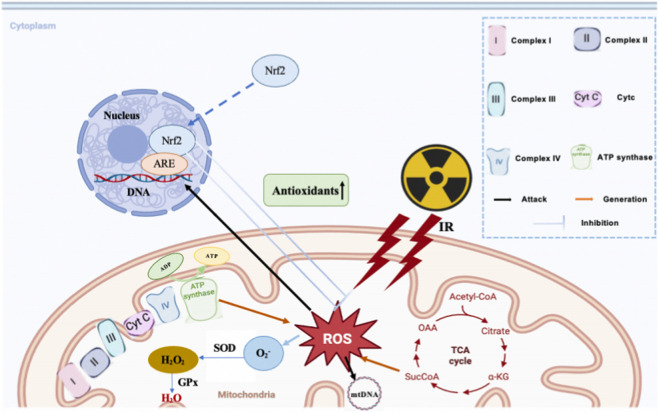
IR-induced mitochondrial ROS amplification and adaptive antioxidant feedback. Ionizing radiation (IR) damages mitochondrial respiratory chain complexes, impairs electron transfer, and increases electron leakage, thereby promoting mitochondrial ROS (mROS) accumulation. Elevated mROS further aggravates mitochondrial dysfunction, including respiratory chain injury, mitochondrial membrane damage, and redox imbalance, forming a self-amplifying oxidative stress loop. In parallel, mROS activates compensatory antioxidant defense systems. Superoxide dismutases (SODs) convert superoxide anions into hydrogen peroxide, which is further detoxified by glutathione peroxidases (GPx), catalase, and related peroxidases. Increased oxidative stress also activates the KEAP1–Nrf2–ARE pathway: oxidative modification of KEAP1 relieves Nrf2 repression, allowing Nrf2 nuclear translocation and ARE-driven transcription of antioxidant and cytoprotective genes. This adaptive antioxidant feedback may protect normal cells from excessive radiation-induced oxidative injury, but in tumor cells it can also buffer RT-induced ROS, support redox homeostasis, and contribute to radioresistance. IR, ionizing radiation; ROS, reactive oxygen species; mROS, mitochondrial ROS; KEAP1, Kelch-like ECH-associated protein 1; Nrf2, nuclear factor erythroid 2–related factor 2; ARE, antioxidant response element; SOD, superoxide dismutase; GPx, glutathione peroxidase; TCA, tricarboxylic acid cycle.

Tumor cells maintain a delicate balance between ROS generation and scavenging, keeping ROS levels within a range that promotes tumorigenesis (Figure 2). On one hand, cancer cells achieve this by upregulating antioxidant proteins to prevent excessive ROS accumulation, thereby sustaining a proliferative state and avoiding ROS-induced cell death triggered by mitochondrial permeability transition (Wang R. et al., 2023). Among these mechanisms, nuclear factor erythroid 2–related factor 2 (Nrf2) is recognized as the central regulator of the antioxidant response. Nrf2 alleviates oxidative stress by binding to antioxidant response elements (AREs) in the promoters of target genes. Upon exposure to mROS, conformational changes occur in its allosteric repressor protein KEAP1, leading to the release of Nrf2 in the cytoplasm. Nrf2 then translocates into the nucleus, where it induces the transcription of genes involved in antioxidant defense (Tossetta and Marzioni, 2022). In addition, studies have shown that Nrf2 can also be activated by the stress response gene p62/SQSTM1 (sequestosome 1) (Shi et al., 2022), further supporting the antioxidant defense of tumor cells.

On the other hand, superoxide dismutases (SODs), as enzymes that catalyze the clearance of superoxide radicals, also play a crucial role (Che et al., 2016). Three types of SODs promote the conversion of superoxide to hydrogen peroxide. Among the three mammalian SOD isoforms, SOD1 is a Cu/Zn-dependent enzyme predominantly localized in the cytosol, but it is also present in the mitochondrial intermembrane space and nucleus (Trist et al., 2021); SOD2 is a Mn-dependent enzyme localized in the mitochondrial matrix (Li et al., 2024a); and SOD3, also known as extracellular SOD, is secreted into the extracellular space and associates with the extracellular matrix (Zelko et al., 2002). These compartment-specific SODs catalyze the dismutation of superoxide anions into hydrogen peroxide and molecular oxygen. Subsequently, hydrogen peroxide is further detoxified by catalase, peroxiredoxins, and glutathione peroxidases, thereby completing the antioxidant defense cascade. Beyond their enzymatic activity, SOD1 has recently been identified as a transcription factor that responds to global increases in intracellular ROS. Upon exposure to hydrogen peroxide, SOD1 translocates to the nucleus, where it binds to promoters and upregulates the expression of genes involved in oxidative stress resistance and repair (Di et al., 2024). All of these scavenging and antioxidant defense systems are critical for counteracting the oxidative radicals and ROS generated by IR. Therefore, the mitochondrial redox hub may represent a highly promising therapeutic target in anticancer strategies.

Beyond direct detoxification of ROS, antioxidant defense systems may also influence the duration and intensity of DNA damage response (DDR) signaling after RT. RT-induced mitochondrial ROS and respiratory chain damage occur in parallel with nuclear DNA damage, thereby activating checkpoint pathways that determine whether cells undergo repair, cell-cycle arrest, checkpoint recovery, or death. When oxidative and genotoxic stress remains within a repairable range, checkpoint activation can provide time for DNA repair and cellular recovery. However, cell-cycle re-entry after DNA damage is not a passive process. Checkpoint recovery is actively regulated by mitotic kinases and phosphatases, including PLK1 and CDC25 family members. CDC25B has been reported to cooperate with PLK1 to promote G2-phase cell-cycle resumption after DNA damage, whereas PLK1 can stimulate cyclin B1–CDK1 activation and contribute to checkpoint silencing. In particular, PLK1-mediated regulation of 53BP1 and CHK2 can attenuate the ATM/CHK2 checkpoint branch, thereby promoting mitotic entry after DNA damage (Verma et al., 2019). Thus, the balance between persistent DDR activation and PLK1–CDC25-driven checkpoint recovery may determine whether RT-induced oxidative stress results in repair and survival or progresses toward irreversible cell death.

Importantly, the biological consequences of RT-induced ROS are highly dependent on their magnitude, duration, subcellular localization, and the adaptive capacity of tumor cells. Acute and high-level ROS accumulation after RT can exceed the antioxidant buffering capacity of tumor cells, thereby promoting DNA damage, mitochondrial membrane depolarization, lipid peroxidation, mitochondrial outer membrane permeabilization, apoptosis, and ferroptosis. In this setting, mitochondrial ROS amplification contributes directly to tumor cell killing and may also facilitate DAMP release and ICD-associated immune activation. However, chronic, moderate, or sublethal ROS exposure can have the opposite effect. Instead of inducing irreversible damage, persistent low-level ROS may function as signaling molecules that activate adaptive survival pathways, including Nrf2-mediated antioxidant transcription, HIF-1α-dependent hypoxia adaptation, NF-κB-associated inflammatory and prosurvival signaling, autophagy/mitophagy-mediated mitochondrial quality control, and metabolic reprogramming. These responses can reduce oxidative injury, restore redox homeostasis, support repair after RT, and ultimately promote radioresistance. Therefore, ROS should not be viewed simply as uniformly beneficial radiosensitizing mediators. Rather, successful redox-based radiosensitization requires pushing tumor cells beyond their adaptive antioxidant threshold while avoiding or concurrently blocking compensatory survival programs.

## 3 Mitochondria-regulated pathways of immunogenic cell death

### 3.1 Hallmarks and initiation of ICD

**FIGURE 3:**
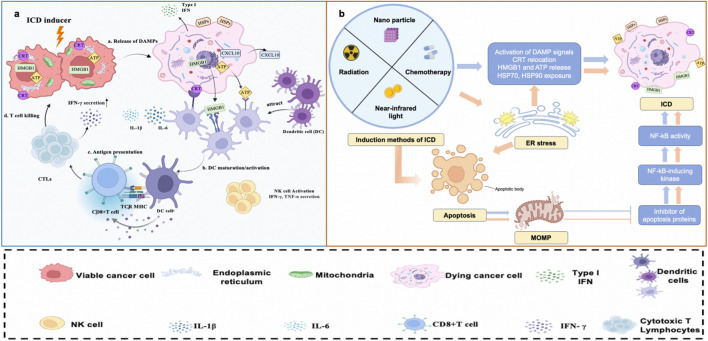
Mitochondria-regulated ICD and inflammatory signaling. **(a)** Mitochondrial damage promotes ICD through mitochondrial ROS accumulation, ER stress, calcium signaling, ATP release, and exposure or release of DAMPs such as CRT, HMGB1, ATP, and mtDNA, thereby enhancing dendritic cell maturation and antitumor immune activation. **(b)** BAX/BAK-mediated MOMP releases cytochrome c and activates caspase-dependent apoptosis. When caspase activity is inhibited or insufficient, MOMP can lead to mitochondrial inner membrane herniation and mitochondrial inner membrane permeabilization, enabling mtDNA release into the cytosol. Cytosolic mtDNA activates the cGAS–STING pathway, induces type I interferon signaling, and promotes NF-κB-dependent inflammatory responses. Therefore, caspase-inhibited MOMP provides a mechanistic link between mitochondrial apoptosis and inflammatory, potentially immunogenic cell death.

ICD is a form of regulated cell death (RCD) induced by various stressors, characterized by the release or exposure of damage-associated molecular patterns (DAMPs) that elicit antitumor immune responses. The major DAMPs include high-mobility group box 1 (HMGB1), calreticulin (CRT), adenosine triphosphate (ATP), and heat shock proteins (HSPs) (Zhu et al., 2021) (Figure 3a). Studies have shown that ICD can restore the immunogenicity of multiple cancers and improve therapeutic efficacy (Galluzzi et al., 2024). Although the mechanisms of ICD are complex, one of the most important hallmark events is the translocation of calreticulin (CRT) from the ER to the surface of dying cells (Kroemer et al., 2022). This process facilitates the uptake and presentation of tumor antigens by antigen-presenting cells, particularly dendritic cells (DCs), thereby effectively initiating tumor-specific immune responses. Promoting factors of CRT translocation include ROS-regulated ER stress arm and PEK-mediated eIF2α protein phosphorylation (Hu et al., 2024). Thus, intracellular ROS production is a prerequisite and foundation for ICD induction (Hu et al., 2024). Based on this, researchers have hypothesized that oxidative stress within specific organelles of tumor cells may play a decisive role in triggering ICD. In 2019, Chen et al. were the first to investigate the link between mitochondrial oxidative stress and ICD, thereby providing initial mechanistic insights into how mitochondrial ROS–mediated oxidative stress can induce ICD in tumor cells and highlighting the close relationship between mitochondria and ICD (Chen et al., 2019). The induction of ICD after RT should also be considered in relation to checkpoint dynamics. RT-induced mitochondrial damage, ROS accumulation, and mtDNA release occur alongside nuclear DNA damage and DDR activation. Therefore, whether a cell proceeds toward ICD-like immune activation depends not only on the magnitude of mitochondrial stress but also on whether checkpoint signaling is sustained or terminated. If DNA damage and mitochondrial dysfunction exceed the capacity for repair, prolonged checkpoint activation may be followed by apoptosis, ferroptosis, mitotic catastrophe, or other forms of regulated cell death accompanied by DAMP release. Conversely, PLK1–CDC25-mediated checkpoint recovery or checkpoint adaptation may permit cell-cycle re-entry despite residual damage, potentially delaying or limiting ICD and contributing to radioresistance. In this context, PLK1 acts not only as a mitotic kinase but also as a checkpoint recovery regulator capable of attenuating ATM/CHK2 signaling and promoting mitotic entry after DNA damage (Verma et al., 2019). This provides a mechanistic link between RT-induced damage sensing, mitochondrial stress, cell-cycle fate, and the timing of immunogenic cell death.

### 3.2 The role of mitochondria in ICD

mtDNA is enriched in unmethylated CpG sequences, which can be recognized by the innate immune system through Toll-like receptor 9 (TLR9) or cytosolic DNA sensors (Fang et al., 2021). When mtDNA leaks from mitochondria into the cytoplasm, or is taken up by immune cells, it can be detected by cyclic GMP–AMP synthase (cGAS), leading to the activation of the cGAS–STING signaling pathway. This in turn induces the production of type I interferons and various inflammatory cytokines, functioning as a classical DAMPs (Gehrke et al., 2013). Moreover, as noted above, mtDNA lacks histone packaging and protection, making mitochondria particularly vulnerable to stress-induced damage such as ROS attack (Caito et al., 2015). Feng et al. demonstrated that, in a PTT/CDT-based nanotherapy model, mitochondrial stress events, including mitochondrial membrane potential (ΔΨm) loss and mROS generation, were associated with subsequent ER stress and disruption of mitochondria-associated ER membranes (MAMs) (Feng et al., 2023). These findings suggest that, in certain experimental contexts, mitochondrial oxidative stress may function as an upstream or amplifying signal for ER stress-associated ICD rather than acting as an independently superior ICD trigger. Consistently, focused mitochondrial oxidative stress has been shown to induce robust ICD in tumor models, while mitochondria–ER crosstalk can further engage the ROS–PERK–eIF2α pathway, thereby promoting DAMP exposure/release, dendritic cell maturation, and antitumor immune activation. Therefore, mitochondrial stress should be viewed as a context-dependent amplifier of ICD, particularly when coupled to ER stress and innate immune sensing pathways (Chen et al., 2019).

During RT, IR enhances mitochondrial oxidative stress and increases membrane permeability, leading to the massive release of mtDNA (Caito et al., 2015). This released mtDNA is often accompanied by prominent oxidative modifications, such as the formation of 8-hydroxyguanosine (8-OHG). Compared with unmodified mtDNA, oxidized mtDNA is more resistant to degradation by the 3′repair exonuclease 1 (TREX1), allowing it to persist longer both intracellularly and extracellularly, and to more effectively activate the STING pathway (Fang et al., 2021). This process not only facilitates the cross-presentation of tumor antigens by DCs but also enhances cytotoxic T lymphocyte (CTL)-mediated tumor-specific immune responses (Illner and Scherthan, 2013). Current studies suggest that one of the reasons why inactivated tumor cell vaccines generated by RT are able to induce stronger antitumor immune effects lies in ROS-induced mtDNA oxidation. Such oxidized mtDNA functions as a natural adjuvant that efficiently activates innate immunity, thereby laying the foundation for subsequent adaptive immune responses (Deng et al., 2014).

### 3.3 Mitochondrial apoptosis and inflammatory signaling

In the mitochondria-mediated apoptotic pathway, various upstream effectors, such as DNA damage, ROS accumulation, cellular stress responses, and multiple death signals, can trigger the initiation of apoptosis. A critical and irreversible event in this process is mitochondrial outer membrane permeabilization (MOMP) (Burke, 2017). MOMP is driven by the oligomerization of BAX (BCL-2 associated X protein) or BAK (BCL-2 antagonist killer 1) on the mitochondrial outer membrane (Burke, 2017). Using super-resolution optical microscopy, Schafer B et al. observed that MOMP manifests as large lipid pores exceeding 100 nm in diameter, which consistently coincide with the release of cytochrome c (cyt c) (Schafer et al., 2009). Once cytochrome C is released, it irreversibly activates caspases, driving the apoptotic process to its conclusion (Vringer and Tait, 2023).

The discovery of the BCL-2 gene spurred research into a family of over 20 related proteins, each possessing four homologous domains (BH1-BH4) (Rühl et al., 2025). During MOMP, channels are formed in the mitochondrial outer membrane that permit the passage of macromolecules, including cyt c (Schafer et al., 2009). Studies have shown that the BAX/BAK-mediated MOMP process is subject to both positive and negative regulation by BCL-2 family proteins. Although multiple competing models have been proposed to explain the precise regulatory mechanisms, both *in vitro* and *in vivo* evidence has consistently demonstrated that MOMP is tightly controlled by the BCL-2 protein family (Srivastava et al., 2025).

Mitochondria-dependent apoptosis is generally considered a non-inflammatory form of cell death, allowing the host to rapidly and efficiently remove cellular debris without triggering an immune response (Winter et al., 2022). However, recent studies have demonstrated that caspase activity is essential for maintaining the non-inflammatory nature of mitochondrial apoptosis (Hall-Younger and Tait, 2025). When caspase activity is inhibited following MOMP, cells still undergo death, but this is accompanied by type I interferon (IFN) responses and NF-κB activation (Figure 3b) (Hall-Younger and Tait, 2025). Riley et al. found that after MOMP, the pores formed by BAX/BAK progressively enlarge, allowing unfolded inner mitochondrial membranes to “herniate” outward. Under caspase-inhibited conditions, these herniated membranes undergo permeabilization, releasing mtDNA into the cytoplasm. This will activate the cGAS–STING signaling pathway and induce IFN synthesis (Riley et al., 2018). This process results in the production of pro-inflammatory cytokines, the initiation of immune responses against dying cells, and potentially the activation of anti-tumor immunity. These findings indicate that mitochondria-mediated apoptosis can acquire immunostimulatory features under specific conditions, particularly when caspase activity is limited or when mtDNA escapes into the cytosol and activates innate immune sensing pathways such as cGAS–STING. Therefore, rather than being uniformly immunogenic, mitochondrial apoptosis may contribute to ICD-like immune activation in a context-dependent manner. This provides a mechanistic framework for understanding how selected therapeutic stresses, including some chemotherapeutic agents and RT, may promote immunogenic signaling when mitochondrial damage, DAMP release, and innate immune activation are sufficiently engaged.

### 3.4 ROS-driven novel cell death pathways

**FIGURE 4:**
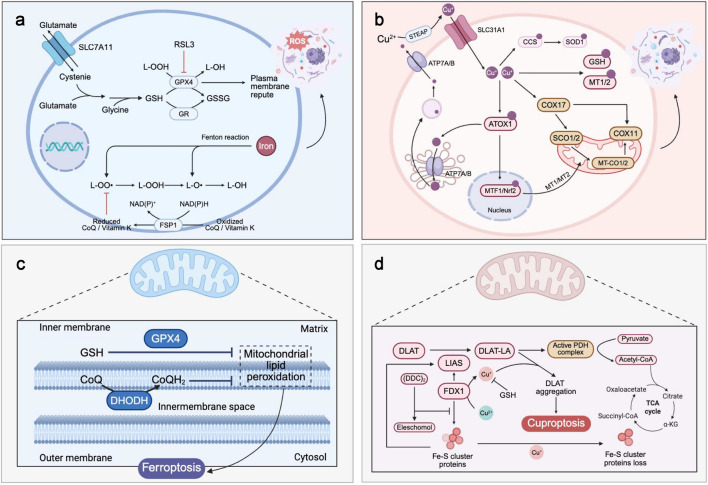
Mechanisms of ferroptosis and cuproptosis in mitochondria. **(a)** Ferroptosis is induced by the accumulation of lipid peroxides, which are generated through the interaction of iron and ROS. Key players in ferroptosis include GPX4, GSH, and SLC7A11. The process is triggered by the inhibition of GPX4, leading to lipid peroxidation and cell death. The year 2012 marks the first description of ferroptosis as an iron-dependent, non-apoptotic form of regulated cell death. **(b)** Cuproptosis is a copper-dependent form of cell death characterized by the accumulation of copper ions, which disrupt Fe-S cluster proteins and interfere with mitochondrial function. Key components involved in cuproptosis include FDX1, DLAT, and the PDH complex, which play critical roles in maintaining mitochondrial integrity. The year 2022 marks the introduction of cuproptosis as a copper-dependent form of regulated cell death. **(c)** Ferroptosis in mitochondria is mediated by lipid peroxidation, where the accumulation of iron and ROS leads to mitochondrial membrane damage and cell death. The process is regulated by mitochondrial enzymes such as GPX4 and transporters like SLC7A11. **(d)** In cuproptosis, copper ions accumulate within the mitochondria, leading to the disruption of mitochondrial function and the loss of Fe-S clusters. This disrupts key mitochondrial processes, including the pyruvate dehydrogenase (PDH) complex, and contributes to cell death. GPX4: glutathione peroxidase 4; GSH: glutathione; SLC7A11: solute carrier family 7 member 11; ROS: reactive oxygen species; Fe-S clusters: iron-sulfur clusters; Cu2+: copper ions; DLAT: dihydrolipoamide S-acetyltransferase; FDX1: ferredoxin 1; PDH: pyruvate dehydrogenase; DLAT-LA: lipoylated DLAT.

In addition to the classical mitochondrial apoptotic pathway, RT-induced ROS can also trigger various other forms of programmed cell death, among which ferroptosis has attracted particular attention. Ferroptosis is an iron-dependent form of cell death driven by excessive lipid peroxidation, and is regarded as a potential type of ICD (Figure 4a). Its morphological hallmarks include reduced mitochondrial volume, increased mitochondrial membrane density, diminished or absent cristae, and outer mitochondrial membrane rupture (Xie et al., 2016). The induction of ferroptosis involves activation of mitochondrial voltage-dependent anion channels (VDAC) and mitogen-activated protein kinases (MAPKs), upregulation of ER stress, and inhibition of the cystine/glutamate antiporter system (Wang et al., 2025). Negative regulators of ferroptosis, such as glutathione peroxidase 4 (GPX4), heat shock protein beta-1 (HSPB1), and Nrf2, function by limiting ROS generation or reducing cellular iron uptake. In contrast, NADPH oxidases and p53 (particularly acetylation-deficient mutant p53) can positively regulate ferroptosis by enhancing ROS production or suppressing expression of the light chain subunit SLC7A11 of the cystine/glutamate antiporter (Wang et al., 2025).

IR severely damages mitochondrial membranes, altering their permeability and causing mitochondrial swelling, vacuolization, and cristae disruption. This damages the electron transport chain (ETC) and leads to ROS accumulation, thereby inducing ferroptosis (Figure 4c). Interference with key regulatory genes of ferroptosis could weaken the antitumor effects of RT (Galy et al., 2024). Moreover, Efimova et al. reported that inoculation of irradiated tumor cells undergoing ferroptosis into immunocompetent mice elicited a certain degree of immune protection against subsequent challenges with live tumor cells. This finding suggests that ROS-induced ferroptosis is not merely a terminal cell death pathway, but may also contribute to the activation of antitumor immunity through the release of DAMPs, thereby establishing a close link with ICD (Efimova et al., 2020).

Copper, as one of the essential trace metal elements in the human body, plays a crucial role in regulating important physiological processes. Previous studies have shown that tumor cells, compared with normal cells, exhibit a significantly increased demand for copper ions, characterized by elevated copper uptake that subsequently activates multiple signaling pathways, including p53 and HIF-1α, thereby promoting tumor proliferation and metastasis (Jiang et al., 2025). In 2022, Tsvetkov et al. proposed the concept of cuproptosis, a novel form of copper-dependent regulated cell death. This process is triggered by copper-mediated aggregation of thioredoxin proteins and the loss of mitochondrial iron–sulfur cluster proteins, leading to proteotoxic stress and ultimately cell death (Figure 4b) (Tsvetkov et al., 2022). Moreover, studies have demonstrated that RT increases cytosolic free copper levels, thereby contributing to the development of radioresistance. Excess copper ions also cause excessive ROS production, disrupt mitochondrial membrane permeability, and activate the mitochondrial apoptotic pathway. This process is usually accompanied by innate immune activation, which enhances the immunogenicity of cell death. Modulating the intracellular distribution of copper, for example, transporting excessive cytosolic copper into mitochondria, may hold therapeutic potential (Figure 4d) (Tian et al., 2023). Such a strategy not only reduces cytosolic copper levels, inhibiting tumor cell proliferation and reversing radioresistance, but also promotes mitochondrial copper accumulation, thereby effectively inducing cuproptosis. Distinct from other forms of RCD, such as apoptosis, ferroptosis, autophagy and pyroptosis, cuproptosis exhibits a unique molecular mechanism (Zhang et al., 2022a; Meng et al., 2025a). Increasing mitochondrial copper levels is expected to enhance the lethal effects of RT and induce ICD, ultimately improving therapeutic outcomes.

In addition to apoptosis and ferroptosis, RT-induced mitochondrial and genotoxic stress can also drive cellular senescence, which represents a non-lethal but durable cell-cycle arrest state. This fate is particularly relevant because senescent tumor cells remain viable and may shape the tumor microenvironment (TME) through the senescence-associated secretory phenotype (SASP), making their immunological consequences highly context-dependent. A useful example of this cell-fate balance is provided by the p21–ASK1 axis. De Blasio et al. demonstrated that p21^Waf1/Cip1^ promotes cell-cycle arrest and senescence while restraining ASK1/p38-mediated apoptosis under stress conditions. In p21-deficient keratinocytes, ASK1/p38 signaling and cleaved PARP1 were increased, whereas combined loss of p21 and ASK1 impaired senescence-like features and reduced apoptosis (De et al., 2021). These findings suggest that p21 and ASK1 cooperate to regulate the balance between senescence and apoptotic elimination of stressed cells. In the context of RT, this axis may help explain how mROS and DNA damage bias tumor cells toward different outcomes: a p21-dominant response may favor cell-cycle arrest and senescence, whereas reduced p21 restraint or enhanced ASK1/p38 activation may shift stressed cells toward apoptosis and, when accompanied by DAMP release and innate immune activation, ICD-like antitumor responses. Therefore, modulation of the p21–ASK1 axis may represent a potential strategy to redirect RT-induced mitochondrial stress from a less directly immunogenic senescent state toward more immunogenic cell death, although this concept requires further validation in irradiated tumor models.

In summary, mitochondria regulate the immunogenicity of cell death through multiple pathways: the release of DAMPs (such as mtDNA and ATP), ROS-triggered stress responses, and the cascade with ER stress (e.g., CRT unfolding requires upstream ROS signaling) collectively determine whether radiation-induced cell death activates the immune system. Understanding how mitochondria influence ICD will aid in developing novel strategies combining RT with immunotherapy.

## 4 Mitochondria-targeted strategies to enhance radiotherapy efficacy and antitumor immune responses

### 4.1 Small-molecule mitochondria-targeted strategies

RT primarily relies on ROS-mediated DNA damage to eliminate tumor cells, and mitochondria serve both as a major source of ROS and as a central hub for integrating cell death signals. Consequently, small molecules designed to amplify mitochondrial ROS, inhibit antioxidant systems, regulate metabolism, or activate apoptotic pathways have emerged as important tools for radiosensitization and immune activation.

ROS amplifiers function by disrupting the electron transport chain or perturbing mitochondrial redox homeostasis to enhance oxidative stress. For example, rotenone and antimycin A inhibit complexes I and III, respectively, leading to superoxide accumulation. Cinnamaldehyde depletes GSH, triggering a burst of ROS; when delivered via nanocarriers, it markedly enhances the efficacy of RT and promotes CD8^+^ T cell infiltration (Gao et al., 2020; Shi et al., 2021; Wan et al., 2023). Moreover, fenofibric acid derivatives elevate mitochondrial ROS by inhibiting complex I, thereby activating ER stress and the cGAS–STING pathway. This approach successfully induces systemic antitumor immune responses in otherwise immunologically “cold” tumor models (Wang Y. et al., 2023). Importantly, the value of these strategies lies not only in amplifying cytotoxicity but also in facilitating the release of DAMPs required for ICD, ultimately improving responsiveness to immune checkpoint blockade (ICB).

However, tumor cells often rely on highly efficient mitochondrial antioxidant systems to counteract RT-induced ROS. Enzymes such as Mn-SOD, GSH, and catalase are frequently overactivated in various tumors, forming a protective barrier against oxidative stress. Small-molecule agents targeting these mechanisms have shown promising progress. For instance, buthionine sulfoximine depletes GSH, while ATN-224 inhibits SOD activity, both promoting ROS accumulation and enhancing radiosensitivity (Cadavid Vargas et al., 2022; Trapp et al., 2009; Lee et al., 2013). In addition, the Nrf2 signaling pathway plays a central role in maintaining redox homeostasis, as its activation upregulates a wide array of antioxidant enzymes (Ghareghomi et al., 2022; Mukherjee and Gopalakrishnan, 2023). Strategies that inhibit Nrf2 activity, such as small-molecule Nrf2 inhibitors, can block the adaptive antioxidant response of tumor cells to RT and thereby further enhance ICD (Li et al., 2024b; Chen et al., 2015).

Building on these findings, researchers have also explored strategies of metabolic reprogramming and oxygen supply modulation. By reducing mitochondrial oxygen consumption to alleviate hypoxia, tumor radiosensitivity can be indirectly improved. Papaverine and its derivatives inhibit complex I, thereby lowering oxygen consumption and significantly enhancing RT efficacy. At low concentrations, MitoQ induces a metabolic shift toward glycolysis, increases tumor oxygen tension, and has demonstrated both efficacy and safety in breast cancer models as well as in a phase I clinical trial (MDA-MB-231/MCF7 xenograft, nude mouse; 5 Gy single dose or 2 Gy × 5 fractions) (Benej et al., 2018; Rondeau et al., 2024; Smith and Murphy, 2011). Unlike traditional exogenous oxygen supplementation, this approach enhances relative oxygen availability by decreasing oxygen demand, making it mechanistically more generalizable.

In addition to metabolic regulation, direct activation of mitochondrial apoptotic pathways represents another important strategy. The Bcl-2 family of proteins regulates MOMP and apoptosis. BH3 mimetics, such as ABT-263 and ABT-199, can relieve the inhibition of pro-apoptotic proteins, thereby inducing cytochrome c release and caspase activation (Tse et al., 2008; Moyer et al., 2025). Novel small molecules such as BKA-073 can directly activate Bak, producing marked synergy with RT in lung cancer models (Park et al., 2021). In parallel, overexpression of IAP suppresses RT-induced apoptotic signaling. SMAC mimetics, including GDC-0152 and birinapant, antagonize IAP to promote immune remodeling and vascular regulation, thereby enhancing CD8^+^T cell infiltration and improving radiosensitization in glioblastoma models (Snacel-Fazy et al., 2024; Cerna et al., 2021). Table 1 summarizes representative small molecules targeting mitochondrial apoptosis pathways, highlighting their mechanisms and synergistic effects with RT in various cancer models.

**TABLE 1:** Representative mitochondrial apoptosis-targeting small molecules in cancer. This table summarizes selected small molecules that modulate mitochondrial apoptotic signaling, including their targets or mechanisms, the specific mitochondrial apoptosis pathways activated, and their reported therapeutic effects. MOMP, mitochondrial outer membrane permeabilization; Cyt c, cytochrome c; RT, radiotherapy; IAP, inhibitor of apoptosis protein; TAMs, tumor-associated macrophages.

Drug	Target/Mechanism	Mitochondrial apoptosis pathway activated	Therapeutic effect/Function
ABT-263/ABT-199/ABT-737	BH3 mimetics; inhibit Bcl-2, Bcl-xL, Mcl-1	Relieves inhibition of pro-apoptotic proteins (BAX/BAK/BH3-only) → MOMP ↑ → Cyt c release → Caspase 3/9 activation	Induces tumor cell apoptosis; enhances radiosensitivity
BKA-073	Direct activator of Bak BH3 domain	BAK conformational change and oligomerization → MOMP ↑ → Cyt c release → Caspase activation	Significant anti-tumor effect; synergizes with RT and Bcl-2 inhibitors
GDC-0152	SMAC mimetic; antagonizes IAP	IAP inhibition relieved → Caspase-3 activation → TAMs pro-apoptotic and pro-inflammatory polarization	Promotes TAMs reprogramming from immunosuppressive to anti-tumor phenotype; enhances CD8^+^ T cell infiltration; suppresses tumoroid growth
Birinapant	SMAC mimetic; antagonizes IAP	Relieves IAP inhibition → Cyt c/Apaf-1/Caspase-9 cascade activation	Induces tumor cell apoptosis; enhances RT.

MOMP, mitochondrial outer membrane permeabilization; Cyt c, cytochrome c; RT, radiotherapy; IAP, inhibitor of apoptosis protein; TAMs, tumor-associated macrophages.

Finally, several other metabolic regulators have also demonstrated potential as adjuncts to RT. Dichloroacetate restores mitochondrial oxidative function by inhibiting pyruvate dehydrogenase kinase, leading to a marked increase in ROS and enhanced radiosensitivity in glioma and breast cancer models (Zwicker et al., 2013; Shen et al., 2020; de Mey et al., 2020). Metformin, through inhibition of complex I and activation of AMPK, alleviates hypoxia and modulates cell cycle progression and DNA repair, making it one of the most extensively studied candidates for RT sensitization (Tsakiridis et al., 2021; Cheng et al., 2016; Sesen et al., 2015; Santos et al., 2024; Li et al., 2025). Overall, due to their well-defined mechanisms and broad clinical applicability, these metabolic agents are emerging as an important direction in the development of RT adjuvant strategies.

Collectively, small-molecule agents enhance radiosensitization through multifaceted interventions in mitochondrial function, ranging from ROS amplification and antioxidant inhibition to metabolic reprogramming and apoptotic activation, while simultaneously exerting immunomodulatory effects. Their combination with nanodelivery systems or genetic tools holds promise for further improving the precision and controllability of treatment.

### 4.2 Nanoparticle-based mitochondrial targeting strategies

Utilizing nanomaterials to achieve precise delivery of radiosensitizers to mitochondria has emerged as an important strategy for improving therapeutic efficacy. A widely adopted approach is to functionalize nanoparticle surfaces with targeting moieties such as triphenylphosphonium (TPP^+^), while encapsulating high-Z elements such as gold, iodine, or bismuth, or radio-responsive agents including Ce6 and Cu-I, to amplify the cytotoxic effects of X-ray irradiation (Yu et al., 2017; Liu et al., 2024). For example, Cu-I@BSA nanoparticles impair mitochondrial function and promote excessive ROS accumulation, thereby synergizing with X-ray irradiation to markedly enhance radiosensitization while maintaining favorable biocompatibility (HepG2 xenograft, nude mouse; 1–2 Gy single fraction) (Ma et al., 2024). Similarly, an X-PDT platform that co-encapsulates a photosensitizer, gold nanoparticles, and TPP within PLGA can induce strong mitochondrial stress under low-dose RT, achieving antitumor efficacy comparable to conventional RT doses with reduced side effects (HCT116 xenograft, scid mouse; 4 Gy single dose) (Deng et al., 2020). Taken together, these findings indicate that mitochondrial-targeted delivery represents a promising strategy for improving RT efficacy while minimizing toxicity.

In addition to directly inducing apoptosis through mitochondrial damage, mitochondrial-targeted nanosystems can further amplify the effects of RT by enhancing ICD. Recent studies have demonstrated that nanoparticle-mediated mitochondrial stress effectively promotes ICD, with key events including ROS burst, Ca^2+^ overload, activation of the cGAS-STING pathway, and collapse of mitochondrial membrane potential (Luo et al., 2023; Wen et al., 2025; Zhong et al., 2025; Ren et al., 2021). Consequently, RT combined with ICD-enhancing strategies, particularly those employing mitochondria-targeted delivery platforms, is emerging as a promising approach to integrate RT with immunotherapy.

Building on these advances, ROS-responsive nano-systems provide an additional approach to amplifying ICD. A representative example is NP-I-CA-TPP, which delivers cinnamaldehyde to induce ROS generation and deplete GSH, thereby disrupting mitochondrial homeostasis and promoting ICD (Wan et al., 2023). Further studies have incorporated synergistic immunomodulatory mechanisms. One self-amplifying platform was designed in which paclitaxel (PTX) induced ROS accumulation to trigger a positive feedback loop of drug release, while co-delivery of the IDO1 inhibitor 1-MT alleviated immunosuppression, ultimately achieving suppression of tumor growth and reduced metastasis in animal models (4T1 syngeneic, immunocompetent) (Song et al., 2023). In contrast, for melanoma, another study employed a “pseudo-stealth” photosensitizer with prolonged circulation and precise mitochondrial targeting, enabling more concentrated and efficient ROS generation upon irradiation, which strongly induced ICD and enhanced the efficacy of ICB (Shi et al., 2025). Overall, mitochondria serve as a central hub for ROS regulation and represent a prime target for multimodal therapeutic strategies. Mitochondria-targeted, ROS-responsive nanoplatforms hold significant promise in tumor immunotherapy, particularly for brain cancers. By coordinating drug release, ICD induction, immunomodulation, and remodeling of the TME, these systems create a feedback-amplified oxidative intervention paradigm. Combining such platforms with ICB or personalized vaccines may further enhance therapeutic outcomes (Wang M. D. et al., 2024; Qiao et al., 2026).

Furthermore, multi-component synergistic nanocarriers have demonstrated increased complexity and therapeutic potential. Metal–organic frameworks (MOFs) can integrate multiple functional components to simultaneously amplify ROS, disrupt mitochondria, and activate immune responses. For example, ZIF-8@MnCO@DOX combines metal ions and chemotherapeutic drugs to synergistically activate the cGAS–STING pathway, enhancing RT-induced immune responses (Hu et al., 2025). Another study constructed a mitochondria-targeted MOF, Th-Ir-DBB, which produces ROS and releases digitonin upon X-ray irradiation. By consuming glucose and GSH, this system intensifies cellular stress, while cholesterol depletion downregulates multiple immune checkpoints, inducing robust antitumor immunity (Zhen et al., 2025). The Hf-TP-SN platform achieves ROS-triggered, on-demand release of SN38, thereby enhancing the synergistic effects of chemoradiotherapy (Xu et al., 2023). Additionally, upconversion nanoparticles (UCNPs) overcome the penetration limitations of photosensitizers; the TPP-UCNPs@MOF-Pt composite induces mitochondrial membrane depolarization and DAMP release, thereby potentiating ICD (Chen Y. et al., 2023). To target antioxidant mechanisms, siRNA carriers have been employed to silence GPX4, SLC7A11, and other genes, blocking ROS clearance and amplifying RT efficacy (Xu et al., 2024; Zhang et al., 2022b; Deng et al., 2022). Typical strategies include pH-responsive mitochondrial delivery of siRNA to inhibit ATP production, combined with GSH depletion by DEM or light-triggered Ce6 release with GPX4 silencing to prevent ROS scavenging.

Overall, mitochondria-targeted nanoplatforms are evolving from single RT-sensitizing agents toward integrated designs that combine ROS modulation, ICD induction, and immune activation. However, several translational barriers must be acknowledged before clinical application can be realized. First, the tumor selectivity of TPP^+^-based mitochondrial targeting remains imperfect *in vivo*; cationic TPP^+^ conjugates are subject to hepatic and splenic accumulation, raising concerns regarding off-target organotoxicity that have not been systematically evaluated in long-term preclinical studies. Second, high-Z elements and metal-based platforms face unresolved questions regarding biodegradation kinetics, renal clearance, and potential chronic accumulation in reticuloendothelial organs. Third, the enhanced permeability and retention effect, which underpins passive tumor accumulation for most nanoparticle platforms reviewed here, is highly variable in human tumors compared with rodent xenografts, and its clinical predictive value remains debated. Fourth, the field currently lacks standardized protocols for RT dose, irradiation timing relative to nanoparticle administration, and dosing schedules, making cross-study comparison difficult and hindering regulatory translation. Future efforts should therefore prioritize active tumor-targeting strategies, rigorous long-term toxicology studies, and the establishment of standardized combination protocols to accelerate clinical translation.

### 4.3 Gene editing and molecular engineering strategies

CRISPR/Cas9-based genome-wide screening has emerged as a powerful tool for identifying mitochondria-regulating genes associated with radiosensitization. Studies indicate that cancer cell reliance on mitochondrial OXPHOS closely correlates with resistance to RT and chemotherapy. Multiple CRISPR screens consistently demonstrate that disrupting mitochondrial function can markedly enhance cancer cell radiosensitivity through ROS accumulation, mitochondrial depolarization, and ATP depletion (Xu et al., 2023; Chen Y. et al., 2023; Xu et al., 2024; Zhang et al., 2022b) (Wang et al., 2022; Zhang J. Z. et al., 2022; Jiang et al., 2022). In non-small cell lung cancer models, loss of the antioxidant enzyme SOD2 leads to a sharp increase in ROS levels and heightened sensitivity to ROS-generating agents, suggesting that ROS detoxification may represent a metabolic vulnerability of tumors (Jiang et al., 2022). Similarly, in gliomas, SOD1 inhibition renders tumor cells more susceptible to hypoxia, nutrient deprivation, and RT-induced stress (König et al., 2024). These findings underscore the pivotal role of mitochondrial antioxidant mechanisms in modulating RT responses. Notably, a recent study revealed a novel mechanism regulating mitochondrial plasticity in glioma stem cells: methylation of PGC1α at K224 (PGC1α K224me) controls mitochondrial biogenesis, allowing GSCs to evade RT-induced cell death. Both upstream signals and downstream effectors of this axis have been elucidated, suggesting that targeting PGC1α K224me may disrupt the metabolic adaptability of GSCs and enhance RT efficacy (Li M. et al., 2024). Taken together, mitochondrial function and antioxidant systems occupy central positions in the regulation of radiosensitization.

Building on this, another strategy focuses on targeting nuclear-encoded mitochondrial regulatory genes to indirectly modulate mitochondrial function. Research in this area has concentrated on two directions. The first involves controlling mitochondrial dynamics; for example, targeting factors such as DRP1 and MFN1 induces mitochondrial dysfunction, thereby altering cellular metabolism and stress responses and enhancing RT efficacy (Li et al., 2021; Li et al., 2023; Xie et al., 2024). The second approach aims to modulate mitophagy and mtDNA release, thereby influencing immune-related pathways (Yu et al., 2020; Li et al., 2022a; Li et al., 2022b; Yu et al., 2024; Wang Z. et al., 2024; Cheng et al., 2020). For instance, nanoparticle-mediated inducers combined with the mitophagy inhibitor Mdivi-1 promote mtDNA release and robustly activate the cGAS-STING pathway, enhancing NK cell and T cell cytotoxicity (Yu et al., 2024). However, recent studies reveal more complex mechanisms: tumor cells can transfer these damaged mitochondria to tumor-infiltrating lymphocytes, causing mitochondrial dysfunction and metabolic dysregulation in T cells, which impairs antitumor immunity (Ikeda et al., 2025). Additionally, in a GBM model, OMA1-induced mitophagy was shown to activate the cGAS-STING pathway while simultaneously upregulating PD-L1, thereby promoting immune evasion (Cheng et al., 2020). Therefore, mitophagy and mtDNA release play a dual role in the tumor immune microenvironment: they serve as a bridge for immune activation but may also facilitate tumor immune escape, highlighting the urgent need for more precise regulatory strategies (Zhu et al., 2024).

Further studies have explored the direct therapeutic application of genetic engineering. For example, knockdown of doublecortin promotes BAX translocation to mitochondria, induces ROS accumulation, and triggers mitochondria-dependent apoptosis, thereby enhancing the sensitivity of gliomas to chemoradiotherapy (Nadeem et al., 2024). Meanwhile, genetic engineering of immune cells, particularly CAR-T cells, has emerged as a critical approach to improving antitumor efficacy in solid tumors. Optimizing mitochondrial metabolism can enhance CAR-T cell adaptability under hypoxic, nutrient-deprived, and highly oxidative environments (Liu et al., 2026a). For instance, overexpression of FOXO1 significantly promotes mitochondrial biogenesis and oxidative metabolism in CAR-T cells, delays T cell exhaustion, and maintains long-term functionality, showing superior benefits compared with factors such as TCF1 or ID3 (Liu et al., 2026a; Doan et al., 2024; Marchal, 2024). Similarly, introduction of the glucose transporter GLUT1 enhances glycolytic and oxidative phosphorylation activity, reinforces memory phenotypes, and reduces the expression of exhaustion markers, thereby improving CAR-T efficacy in both solid and hematologic tumor models (Shi et al., 2024; Guerrero et al., 2024).

Despite the potential of gene editing and molecular engineering to enhance tumor radiosensitization and immune function, clinical translation faces multiple challenges. First, off-target effects remain a major safety concern (Gao and Chen, 2022). Targeting mitochondrial pathways may inadvertently affect normal metabolic genes, leading to systemic toxicity or functional impairment. Second, mitochondria are widely present across diverse cell types, and non-specific interventions can provoke aberrant responses or immune reactions. Future strategies should focus on developing tissue-specific delivery systems and exploring multi-gene synergistic modulation, such as concurrently targeting metabolism, apoptosis, and autophagy, to enhance efficacy and delay resistance (Meng et al., 2024a; Guo et al., 2024). With the maturation of CRISPR and *in vivo* editing technologies, precise and reversible mitochondrial regulation holds promise as a novel approach for combined RT and immunotherapy, yet preclinical studies must systematically validate long-term safety and individualized applicability.

However, the clinical translation of these strategies must also contend with inherent immunological risks. Tumor cells can transfer damaged mitochondria to CD8^+^ T cells via tunneling nanotubes and extracellular vesicles, impairing T cell oxidative phosphorylation and accelerating exhaustion (Borcherding et al., 2022). Moreover, mitophagy-driven cGAS–STING activation can simultaneously upregulate PD-L1, as demonstrated in GBM models, potentially undermining net immune activation (Cai et al., 2025). Off-target mitochondrial perturbation in normal tissues further raises concerns regarding systemic toxicity. To mitigate these risks, combining mitophagy-activating approaches with concurrent PD-1/PD-L1 blockade, staggering ICI initiation after RT to reduce overlap with peak PD-L1 induction, and preferring transient gene-editing modalities over permanent editing are practical strategies worthy of prospective evaluation.

### 4.4 Bioregulatory and immuno-combinatorial strategies

Beyond small molecules and gene editing tools, biopolymers such as mitochondria-targeting peptides and protein-engineered therapeutics exhibit unique advantages in modulating tumor fate and remodeling the immune microenvironment. Their high structural specificity allows precise engagement with mitochondrial targets, effectively intervening in energy metabolism, oxidative stress, and cell death pathways. In the context of combined RT and immunotherapy, these agents not only induce ICD but also regulate redox homeostasis and immune cell infiltration. For instance, the cell-penetrating peptide RL2 localizes to the mitochondrial membrane, inhibits the respiratory chain, depletes ATP, and activates both autophagy and ICD (Richter et al., 2020; Wohlfromm et al., 2020). To overcome therapeutic resistance, dual-targeting peptide-drug conjugates have been developed, leveraging mitochondrial localization to enhance efficacy while circumventing DNA repair-mediated tolerance (Zhu et al., 2019). Addressing tumor metastasis, therapeutic peptides derived from the C-terminal region of BAX, such as CT20p, target cancer cell mitochondria, disrupt the cytoskeleton, and inhibit cell migration and invasion (Le et al., 2014). In summary, mitochondria-targeted peptides combine apoptosis induction, immune activation, and resistance reversal, highlighting their potential for integration with RT and immunotherapy.

Non-coding RNA regulation has increasingly emerged as a focal point in recent research. miRNAs enhance mitochondria-dependent apoptosis and radiosensitization by downregulating anti-apoptotic proteins such as Bcl-2 and Mcl-1 (Darvish et al., 2023; Li et al., 2017a). They also contribute to metabolic reprogramming; for example, exosomal miR-503-3p induces a shift from OXPHOS to glycolysis in breast cancer cells, accelerating malignant progression (Huang et al., 2021). Furthermore, miR-34a and miR-30a-5p inhibit LDHA to reduce lactate production, while miR-16-1-3p targets PGK1 to suppress glucose metabolism (Li et al., 2017b; Sharma et al., 2022; Ye et al., 2020). Overall, these studies demonstrate that miRNAs exert multidimensional regulation over mitochondrial function, apoptotic pathways, and metabolic networks, directly influencing cell fate and indirectly modulating treatment sensitivity. Similarly, certain long non-coding RNAs regulate mitochondrial function via a molecular sponge mechanism, thereby affecting RT responsiveness. For instance, HOTAIRM1, OTUD6B-AS1, and LOC401312 modulate ROS or ferroptosis to alter radiosensitization (Ahmadov et al., 2021; Zhang et al., 2023; Cao et al., 2025; Tang et al., 2023). Mitochondria-derived circular RNAs such as mcPGK1 inhibit OXPHOS and promote glycolysis through miRNA sponging (Ren et al., 2023; Chen Z. et al., 2023). These findings collectively highlight the central role of ncRNAs in apoptosis, ROS response, metabolic reprogramming, and immune microenvironment regulation, providing novel targets for RT sensitization.

In addition, the TME and systemic metabolic status are key determinants of radiosensitization (Meng et al., 2024b). Local hyperthermia and metabolic interventions can enhance RT efficacy by modulating mitochondrial function. Hyperthermia improves tumor oxygenation and, via the HIF1-α pathway, suppresses mitochondrial oxygen consumption, promotes ROS generation, and increases immune cell infiltration, thereby amplifying radiation-induced damage (Vaupel et al., 2023; Le Guevelou et al., 2022). Repeated mild hyperthermia can sustain blood flow and oxygenation, while combining with nanotechnology-enabled localized heating further elevates ROS levels and achieves tumor-specific mitochondrial modulation (Griffin et al., 2010). Metabolic interventions, such as ketogenic metabolic therapy (KMT), exploit mitochondrial defects and impaired ketone utilization in brain tumor cells to restrict glycolytic substrates and inhibit tumor growth (Seyfried et al., 2008). Preclinical studies suggest that KMT combined with RT enhances antitumor efficacy, and the recently proposed glucose-ketone index provides a feasible tool for precise monitoring of mitochondrial metabolic status (Table 2) (Dur et al., 2024). Overall, hyperthermia regulates oxygen consumption and ROS, whereas KMT restricts glycolysis through substrate competition, both converging on mitochondrial function as the central target, laying the groundwork for future metabolism–physical radiosensitization strategies.

**TABLE 2:** Overview of clinical evidence evaluating metabolic modulation as a radiosensitizing strategy. This table summarizes clinical studies investigating the use of metabolic modulation, particularly ketogenic diets (KD), as a radiosensitizing strategy in cancer treatment. It highlights the key interventions, outcomes, and findings from various trials, demonstrating the impact of metabolic therapies on radiotherapy efficacy across different cancer types. MCT: medium-chain triglyceride; KD: ketogenic diet; PET: positron emission tomography; FDG: [Fluorine-18] 2-deoxy-2-fluoro-D-glucose; MGMT: O^6^-methylguanine-DNA methyltransferase; HBOT: hyperbaric oxygen therapy; PEG: percutaneous endoscopic gastrostomy.

ClinicalTrials.gov ID	Year	Sample size	Study design	Study population	Intervention	Main results	References
—	1995	2	Case report	Pediatric patients with advanced malignant astrocytoma	60% MCT-based KD for 8 weeks combined with standard therapy (radiotherapy and chemotherapy)	PET scans showed an average 21.8% reduction in tumor FDG uptake	Neb et al. (1995)
—	2010	1	Case report	65-year-old woman with MGMT-methylated glioblastoma after subtotal resection	Metabolic therapy consisted of water-only fasting followed by a calorie-restricted 4:1 KD (∼600 kcal/day) maintained throughout chemoradiation, accompanied by vitamin/mineral supplementation, steroid withdrawal	Marked metabolic and radiographic tumor regression during calorie-restricted KD, with recurrence emerging after cessation of strict dietary therapy	Zuccoli et al. (2010)
NCT01419483	2017	9	Phase 1, single arm	Patients with locally advanced non–small cell lung cancer or pancreatic cancer	Oral KD for 6 weeks (non–small cell lung cancer) or 5 weeks (pancreatic cancer) of chemoradiation	Tolerability was poor, with frequent early discontinuation and multiple dose-limiting toxicities	Zahra et al. (2017)
—	2018	1	Case report	38-year-old man with confirmed glioblastoma	Metabolic therapy (72-h fast + KD) with concurrent radiotherapy, temozolomide, adjunct metabolic agents, and HBOT, without steroids	Marked metabolic response and clinical improvement: reduced glucose, elevated ketones, normalized metabolic biomarkers, seizure resolution, improved neurological deficits, and radiologic tumor regression	Elsakka et al. (2018)
—	2019	29	Retrospective cohort study	Patients with grade II–IV astrocytoma (including 19 glioblastoma cases)	Modified Atkins diet during standard chemoradiotherapy	Among glioblastoma patients, 58% developed pseudoprogression, suggesting a potential radiosensitizing effect	Woodhouse et al. (2019)
NCT01754350	2020	50	Randomized, controlled, open-label clinical trial	Patients with recurrent malignant glioma undergoing re-irradiation	KD with intermittent fasting (KD-IF): 3 days of calorically restricted KD (21–23 kcal/kg/day), followed by 3 days of fasting, then 3 days of KD; Control: calorically unrestricted diet	KD-IF did not improve the primary endpoint of PFS6 compared with the control diet	Voss et al. (2020)
NCT02516501	2021	49	Controlled clinical trial	Patients with non-metastatic rectal cancer undergoing curative radiotherapy	KD: KD (natural-food-based KD) during radiotherapy; Sandard diet: Standard diet (unspecified) during radiotherapy	Compared with the standard diet, KD led to significant weekly reductions in body weight and fat mass during radiotherapy, while preserving skeletal muscle mass	Klement et al. (2021)
NCT01975766	2021	12	Phase 1, single arm	Patients with locally advanced head and neck squamous cell carcinoma	KD (KetoCal® via PEG tube, plus allowed water, sugar-free drinks, dietitian-approved foods) combined with radiotherapy and cisplatin-based chemotherapy	The KD showed poor overall tolerability, leading to early dropout in most patients	Ma et al. (2021)

MCT: medium-chain triglyceride; KD: ketogenic diet; PET: positron emission tomography; FDG: [Fluorine-18] 2-deoxy-2-fluoro-D-glucose; MGMT: O^6^-methylguanine-DNA, methyltransferase; HBOT: hyperbaric oxygen therapy; PEG: percutaneous endoscopic gastrostomy.

Given that the ultimate goal of ICD is to mobilize the immune system against residual tumors, bioregulatory strategies are often combined with immunotherapy. Mitochondrial dysfunction represents a key factor in resistance to ICB therapy, and targeting critical mechanisms can reverse T cell exhaustion and improve ICB efficacy (Yang et al., 2024; Meng et al., 2025b; Liu et al., 2026b). Tumor mitochondrial DNA (mtDNA) is among the most frequently mutated regions in the cancer genome. Engineering mtDNA mutations, such as Mt-Nd5, can shift tumor metabolism toward the Warburg effect, remodel the TME, and activate neutrophil-dominated antitumor immune responses, thereby enhancing tumor radiosensitization to ICB (Mahmood et al., 2024). Additionally, disruption of mitochondrial-associated protein PGAM5 in tumor cells modulates mtDNA stress signaling, influencing the polarization and infiltration of M2 immunosuppressive macrophages in the TME and impacting immune evasion and therapeutic outcomes (Wei et al., 2025). These strategies demonstrate that modulating mitochondrial function in tumors can reshape the TME, revealing key nodes across distinct regulatory mechanisms.

Recent studies have also focused on metabolic regulation in immune effector cells. Farsani and colleagues showed that activation of pyruvate kinase M2 (PKM2) enhances mitochondrial function and cytotoxicity in CD8^+^ T cells. By modulating one-carbon metabolism, PKM2 agonists promote mitochondrial biogenesis, improve antitumor capacity, and synergize with anti-PD-1 therapy while reducing immunosuppressive regulatory T cells, highlighting the potential of targeting mitochondrial metabolism to improve immunotherapy responses (Mortazavi Farsani et al., 2025). Together, these findings underscore the central role of mitochondria and associated genes in regulating tumor metabolism, the immune microenvironment, and effector T cell function, providing novel molecular targets and strategies to overcome ICB resistance and optimize combined immunotherapy regimens.

Based on this rationale, metabolic interventions have also been explored to enhance the efficacy of combined RT-immunotherapy. Animal studies demonstrate that a ketogenic diet can improve tumor control and survival outcomes following RT (Allen et al., 2013), and synergistically inhibit tumor growth with anti-PD-L1 antibodies in renal cancer models (Richard et al., 2024). Several ongoing clinical trials (NCT06391099, NCT06896552, NCT03454282, NCT04833439) are evaluating the translational potential of these strategies. In aggregate, these findings indicate that biopolymers, ncRNAs, hyperthermia, and KMT can all enhance radiosensitization through mitochondrial modulation, and their integration with immune checkpoint inhibitors provides a promising avenue for precision combinatorial therapy.

Despite strong preclinical rationale, several clinical trials of mitochondria-targeting metabolic strategies have yielded neutral or negative results. Metformin failed to improve locoregional control in randomized RT trials, likely due to insufficient intratumoral drug concentrations at standard oral doses and lack of patient stratification by metabolic phenotype (Kordes et al., 2015). Ketogenic metabolic therapy, while promising preclinically, has been limited clinically by poor tolerability, weight loss, and high dropout rates in patients undergoing concurrent chemoradiotherapy (Martin-McGill et al., 2017). These findings highlight recurring translational gaps: predominant use of immunodeficient xenograft models, heterogeneity in RT fractionation, and absence of validated predictive biomarkers. Future trials should incorporate metabolic patient stratification and prospective biomarker co-enrollment alongside the ongoing trials listed in Table 2. At present, the clinical evidence summarized in Table 2 remains largely exploratory. Prospective randomized trials with adequate statistical power are required before metabolic intervention can be considered an established radiosensitizing strategy.

## 5 Future perspectives and conclusion

RT exerts effects that extend far beyond local tumor cytotoxicity, profoundly influencing redox homeostasis, metabolic reprogramming, and antitumor immunity. Within this context, mitochondria occupy a central position, serving not only as a major source and target of ROS, but also as a critical hub linking oxidative stress, regulated cell death, and immune activation. As discussed throughout this review, mitochondrial dysfunction can shape the response to RT through multiple interconnected processes, including amplification of ROS signaling, modulation of antioxidant defenses, induction of ICD, release of mitochondria-derived danger signals, and remodeling of the tumor immune microenvironment.

These mechanistic insights provide a strong rationale for mitochondria-targeted radiosensitization. Small molecules, nanoplatforms, gene-editing strategies, and bioregulatory interventions each offer distinct yet complementary means of enhancing RT efficacy. On the one hand, these approaches can intensify oxidative stress, disrupt mitochondrial homeostasis, and promote tumor cell death; on the other hand, they can amplify ICD, facilitate DAMP release, activate innate immune sensing pathways such as cGAS–STING, and improve the responsiveness of tumors to immunotherapy. In this sense, mitochondria represent not merely a metabolic organelle, but a convergent therapeutic node connecting radiotherapy, cell death regulation, and antitumor immunity.

Encouragingly, several mitochondria-targeted approaches have entered early translational evaluation. Strategies aimed at alleviating hypoxia, increasing mitochondrial DNA damage, or enhancing ICD are beginning to show potential in preclinical and early clinical settings. These advances suggest that mitochondria-targeted interventions may be especially valuable for overcoming hypoxia-associated radioresistance, adaptive antioxidant defenses, and immune-cold tumor phenotypes.

Nevertheless, substantial challenges remain before these strategies can be broadly translated into clinical practice. A major priority is to improve tumor specificity while minimizing injury to normal tissues, given the essential role of mitochondria in healthy cells. Additional hurdles include achieving precise and controllable delivery of nanomedicines and gene-editing tools, defining the optimal timing and sequencing of combination regimens, and establishing reliable biomarkers to monitor mitochondrial ROS, ICD induction, and immune activation in real time. Furthermore, because mitochondrial stress can exert context-dependent effects on both tumor cells and immune cells, future studies should more carefully dissect the bidirectional interactions among mitochondrial signaling, nuclear responses, and the metabolic fitness of macrophages and T cells within the TME.

Future progress in this field will likely depend on three major directions. First, the development of imaging and molecular monitoring approaches capable of dynamically visualizing mitochondrial function, ROS burden, and ICD-related events *in vivo* will be essential for precision therapy. Second, predictive biomarkers integrating metabolic, redox, and immune features should be established to identify patients most likely to benefit from mitochondria-targeted radiosensitization. Third, next-generation therapeutic platforms, including TME-responsive nanocarriers, reversible or tissue-selective gene-editing systems, and rational combinations with immune checkpoint blockade, should be designed to maximize efficacy while limiting off-target toxicity.

In conclusion, mitochondria-targeted strategies are reshaping the conceptual framework of radiotherapy from a purely DNA-damage-based modality toward an integrated approach that also exploits metabolic vulnerability and immune activation. By linking ROS regulation to ICD and radioimmunotherapy, these strategies offer a promising route toward more precise, effective, and personalized cancer treatment.
